# The Incidence of Postoperative Complications after Gastrectomy Increases in Proportion to the Amount of Preoperative Visceral Fat

**DOI:** 10.1155/2019/8404383

**Published:** 2019-12-16

**Authors:** Kazuyuki Okada, Tatsuto Nishigori, Kazutaka Obama, Shigeru Tsunoda, Koya Hida, Shigeo Hisamori, Yoshiharu Sakai

**Affiliations:** ^1^Department of Surgery, Graduate School of Medicine, Kyoto University, 606-8507, 54 Kawahara-cho, Syogoin, Sakyo-ku, Kyoto, Japan; ^2^Patient Safety Unit, Kyoto University Hospital, 606-8507, 54 Kawahara-cho, Syogoin, Sakyo-ku, Kyoto, Japan

## Abstract

**Background:**

Visceral obesity is a risk factor for complications after gastrectomy in patients with gastric cancer. However, it is unclear whether postoperative complications decrease with preoperative reduction of visceral fat without the achievement of a nonobese state. This is because previous studies have performed categorical comparisons of obesity and nonobesity. The current study was performed to estimate the impact of the preoperative visceral fat area (VFA) as a continuous variable on postoperative complications after gastrectomy.

**Methods:**

Consecutive patients with gastric cancer who underwent curative gastrectomy between June 2006 and August 2017 at the Kyoto University Hospital were included in this retrospective study. The VFA at the level of the umbilicus was measured using preoperative computed tomography. The relationship between postoperative complications and VFA was investigated with univariate and multivariate analyses.

**Results:**

total of 566 patients were included in the study. Their mean VFA was 110 ± 58 cm^2^, and postoperative complications occurred in 121 patients (21.4%). The larger the VFA (<50, 50–99, 100–149, and ≥150 cm^2^), the higher the incidence of postoperative complications (11%, 14%, 21%, and 38%, respectively, *P* < 0.001). Multivariate logistic regression analyses showed that the VFA was associated with postoperative complications (odds ratio: 1.009, 95% confidence interval (CI): 1.004–1.013, *P* < 0.001), with an incidence of postoperative complications that was 9% (95% CI: 4%–12%) higher for every 10 cm^2^ increase in the VFA.

**Conclusion:**

The incidence of postoperative complications after gastrectomy increases in proportion to an increase in the preoperative VFA.

## 1. Introduction

Gastrectomy for gastric cancer is an invasive surgery and may involve critical complications, such as anastomotic leakage and pancreatic fistula [1]. Preoperative risk stratification is important to define appropriate treatment strategies, and risk reduction via preoperative intervention may decrease the incidence of postoperative complications.

Visceral obesity has been associated with complications after gastrectomy in patients with gastric cancer [2–4]. In these reports, visceral obesity is usually defined as a visceral fat area (VFA) ≥100 cm^2^ measured using computed tomography (CT) [2–7]. The VFA can be reduced over a short preoperative period, unlike risk factors, such as age, sex, comorbidity, and cancer stage [8, 9]. Therefore, preoperative intervention for patients with high VFA may be effective for decreasing the incidence of postoperative complications. However, it is unclear if complications decreased with preoperative reduction in visceral fat without the achievement of a nonobese state because categorical comparison of obesity and nonobesity has been performed in most studies.

The aim of this study was to estimate the impact of preoperative VFA as a continuous variable on complications after gastrectomy in patients with gastric cancer. Using a multivariate model adjusted for confounding factors, we examined whether the incidence of postoperative complications increased in proportion to an increase in preoperative VFA.

## 2. Materials and Methods

### 2.1. Design

A retrospective cohort study was conducted at the Department of Surgery, the Kyoto University Hospital, Kyoto, Japan. The study protocol was approved by the Ethics Committee of Kyoto University Hospital.

### 2.2. Patients

Patients with histologically proven clinical stage I-III gastric cancer who underwent gastrectomy between June 2008 and October 2017 at the Kyoto University Hospital were enrolled in the study from a prospectively maintained database. As per the exclusion criteria, patients who underwent partial gastric resection; proximal gastrectomy; gastroesophagectomy; or simultaneous resection of other organs, except the gallbladder or spleen; or lacked data of CT scan within 2 months before the operation were excluded from the study.

### 2.3. Measurement of VFA

**Figure 1:**
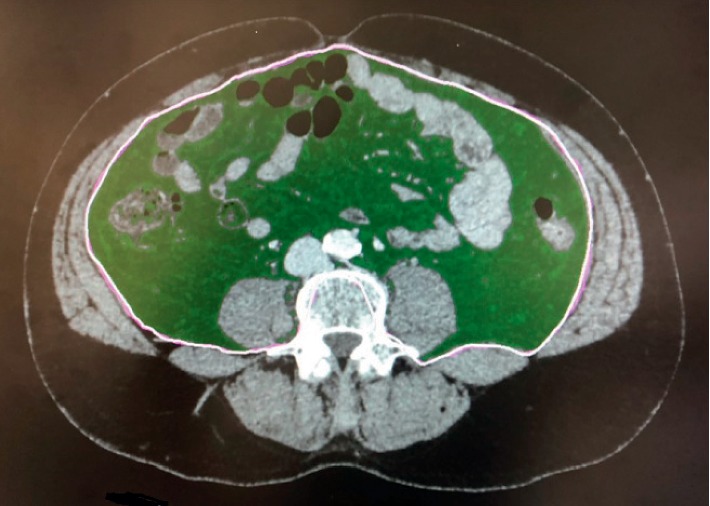
Area of visceral fat at the level of the umbilicus (green field) measured on preoperative computed tomography with AquariusNET server.

The cross-sectional VFA at the level of the umbilicus was retrospectively measured using the most recently performed preoperative CT scan [10–12]. Fat was distinguished from other tissues by measuring the Hounsfield units (HU) on AquariusNET Server (TeraRecon, Foster City, CA, USA). A range of −190 to −30 HU was used to define fat [10]. Manual adjustment was performed by an observer blinded to the postoperative status during VFA assessment (Figure 1). We did not define specific VFA thresholds, and VFA was used as a continuous variable in the multivariate analyses. However, subjects were categorized into the following four groups according to their VFA, <50, 50–99, 100–149, and ≥150 cm^2^ to evaluate the associations with other variables.

### 2.4. Endpoints

The primary endpoint was the incidence of postoperative complications of grade II or higher using the Clavien–Dindo classification [13]. The secondary endpoints included the operating time, blood loss, and length of postoperative hospital stay. Pancreatic fistula was defined as a drain output of any measurable volume of fluid on or after postoperative day 3 with the amylase level in the drainage fluid being three times greater than the serum amylase level, using the definition of the International Study Group on Pancreatic Fistulas [14]. Without the detection of high amylase content, a deep surgical site infection (SSI) was classified as an intra-abdominal abscess.

The relationship between postoperative complications and VFA as a continuous variable was investigated using univariate and multivariate analyses. Potential confounding variables with *P* < 0.2 in the univariate analysis were included in the multivariate analyses. Independent variables were selected from among age (continuous), sex, subcutaneous fat (cm^2^, continuous), BMI (<25 or ≥ 25 kg/m^2^), American Society of Anesthesiologists (ASA) score (≤2 or ≥ 3), diabetes mellitus (yes or no), sarcopenia (yes or no), neoadjuvant chemotherapy (yes or no), clinical stage (cStage, I, II, or III), preoperative albumin (≤3.5 or >3.5 g/dL), operative procedure (distal or total gastrectomy), surgical approach (open, laparoscopic, or robotic), and lymph node dissection (≤D1+ or D2).

Sarcopenia was defined based on the study of Prado et al. [15]. We measured the cross-sectional skeletal muscle mass at the level of the third lumbar vertebra (L3). A threshold range of −29 to 150 HU was used to define the muscle, and hand adjustment of the selected area was performed. The sex-specific cutoffs were 52.4 cm^2^/m^2^ for men and 38.5 cm^2^/m^2^ for women [15]. Tumors were staged using the Japanese Classification of Gastric Carcinoma, 3rd English Edition [16]. After obtaining informed consent, neoadjuvant chemotherapy (S-1 plus cisplatin with or without docetaxel) was administered to patients diagnosed with marginally resectable advanced gastric cancer. Information on patients was extracted from the database, and detailed information was obtained from the original medical records.

### 2.5. Surgical Procedures

The surgical strategy and extent of lymph node dissection were determined according to the Japanese Gastric Cancer Treatment Guidelines, 3rd English Edition [17]. D1+ and D2 lymphadenectomy were indicated for tumors diagnosed as cT1N0 and cN + or cT2-T4, respectively. The indication for laparoscopic gastrectomy had been T3(SS)N0 or earlier gastric cancer. In 2009, a prospective study for more advanced gastric cancer was launched to examine the safety of laparoscopic gastrectomy. Thereafter, open surgeries have been conducted only for patients with a history of upper abdominal surgery, and laparoscopic surgery has been our standard treatment option. The use of the da Vinci surgical system (Intuitive Surgical, Sunnyvale, CA, USA) was limited because it was not covered by public health insurance during the study period. All the procedures were performed or supervised by surgeons certified by the Japan Society for Endoscopic Surgery or a board with equivalent qualifications [18]. Details of our procedures have been reported elsewhere [19–22].

### 2.6. Perioperative Management

Although the patients received nutrition counseling from a registered dietitian, we did not provide special preoperative interventions, such as exercise or nutritional therapy, during the study period. Patients had early postoperative ambulation, water intake from POD (postoperative day) 1, and food intake from POD3.

### 2.7. Statistical Analyses

Continuous variables are shown as mean and standard deviation or as median and range values. Categorical data are expressed as numbers and proportions. The characteristics of the study population in the four VFA categories were analyzed using one-way analysis of variance or chi-square test. In the univariate and multivariate analyses, associations between postoperative complications and the variables were analyzed with logistic regression analysis. Associations of surgical outcomes (operating time, amount of blood loss, and length of postoperative hospital stay) with the VFA were analyzed using multiple linear regression models. The threshold for significance was *P* < 0.05. All the analyses were conducted using JMP pro 13 (ver. 13, SAS Institute, Milan, Italy).

## 3. Results

### 3.1. Patient Characteristics

**Figure 2:**
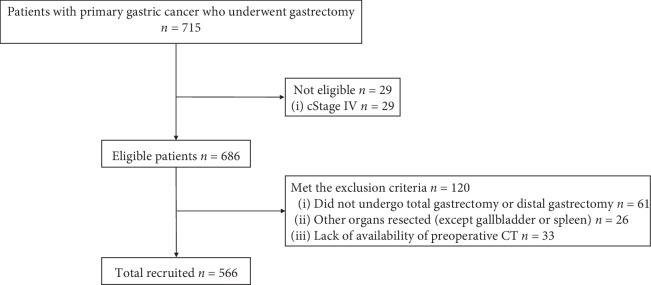
Flow diagram representing patient selection.

Patient selection flowchart is shown in Figure 2. A total of 566 patients were included in the study. The clinical characteristics of the patients in each VFA category are summarized in Table 1. There were significant associations of the VFA categories with age, sex, BMI, ASA score, diabetes mellitus, sarcopenia, neoadjuvant chemotherapy, clinical stage, preoperative albumin, and surgical approach. Of the 566 patients, 81.6% and 94.2% underwent CT scan within one month and 45 days before the surgery.

### 3.2. Relationship between the VFA and Postoperative Complications

**Figure 3:**
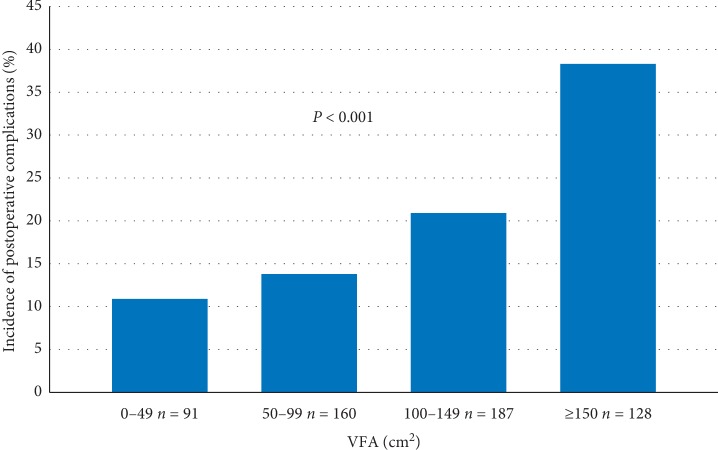
Association between visceral fat area (VFA) categories and postoperative complications (Clavien–Dindo classification grade II or higher). VFA was categorized as <50, 50–99, 100–149, and ≥150 cm^2^.

The overall incidence of postoperative complications was 21.4%. The relationship between the VFA categories and complications is shown in Figure 3. The larger the VFA (<50, 50–99, 100–149, and ≥150 cm^2^), the higher the incidence of complications (11%, 14%, 21%, and 38%, respectively, *P* < 0.001). The relationship between the VFA categories and postoperative complications is shown in Table 2. The major complications were abdominal abscess (8.3%), pancreatic fistula (3.0%), and anastomotic leakage (3.0%). The incidence of abdominal abscess and pancreatic fistula rose with an increase in the VFA (*P*=0.0005 and 0.0089, respectively).

### 3.3. Univariate and Multivariate Analyses of Complications

The results of univariate and multivariate analyses are shown in Table 3. The VFA as a continuous variable was significantly associated with the incidence of postoperative complications (odds ratio (OR): 1.010, 95% confidence interval (CI): 1.007–1.014, *P* < 0.001). Nine variables with *P* < 0.2 in the univariate analysis were selected as explanatory variables in the multiple logistic regression analyses. The VFA (OR: 1.009, 95% CI: 1.004–1.013, *P* < 0.001) and total gastrectomy (OR: 1.79, 95% CI: 1.15–2.79, *P*=0.010) were significantly associated with an increased risk of postoperative complications after adjusting for the confounding variables.

### 3.4. Relationship between the VFA and Surgical Outcomes

The VFA was not significantly associated with the length of postoperative hospital stay in the multivariate analysis (regression coefficient (*β*): 0.008, 95% CI: −0.010–0.027, *P*=0.38). However, the VFA was an independent predictor of the operating time (*β*: 0.23, 95% CI: 0.11–0.35, *P* < 0.001) and amount of blood loss (*β*: 0.43, 95% CI: 0.12–0.73, *P*=0.006). The detailed results of these analyses are shown in Tables [Supplementary-material supplementary-material-1]–[Supplementary-material supplementary-material-1] in the Supplementary Material.

## 4. Discussion

Excess visceral fat is known to be associated with metabolic syndrome and a risk of serious diseases, such as type 2 diabetes [23–26]. In this study, a higher amount of visceral fat was significantly related to known risk factors for complications following gastrectomy, such as advanced age, higher ASA score, diabetes mellitus, and hypoalbuminemia. The standard cutoff value of obesity (VFA = 100 cm^2^) was a good predictor for the incidence of pancreatic fistula (Table 2). However, the incidence of total complications gradually increased in steps in proportion to the VFA as shown in Figure 3. VFA was an independent risk factor for the incidence of complications after gastrectomy after adjusting for the confounding variables. The risk difference between severe (VFA 200 cm^2^) and slight (VFA 120 cm^2^) obesity was estimated by calculating an adjusted OR for VFA of 1.009 (95% CI: 1.004–1.013, *P* < 0.001), indicating that the incidence of postoperative complications was about 9% (95% CI: 4%–12%) higher for every 10 cm^2^ increase in the VFA. We were able to calculate the specific impact of VFA on complications following gastrectomy by treating VFA as a continuous variable. The result indicates that preoperative reduction in VFA even without reaching a nonobese state may contribute to a decrease in the postoperative complications. We believe that the results are also very meaningful for developing a preoperative intervention aimed at reducing the VFA for obese patients with gastric cancer.

There were correlations between VFA and BMI or DM. We conducted stratified analyses for BMI and DM (Tables [Supplementary-material supplementary-material-1] and [Supplementary-material supplementary-material-1] in the Supplementary Material). These analyses also showed significant relationships between VFA and postoperative complications.

In patients with gastric cancer, it is important to reduce postoperative morbidity, the prevalence of which is 20%–45% after gastrectomy [1, 3, 22, 27, 28]. Postoperative complications also lead to poor quality of life and overall survival [28–30]. The present study suggests that a preoperative intervention aimed at reducing visceral fat might be effective for decreasing these complications. However, thus far, few studies have examined the safety and efficacy of preoperative intervention in obese patients with gastric cancer. Inoue et al. found that a very low-calorie diet preoperatively without exercise therapy reduced the body fat mass in patients with early gastric cancer. In this study, one of the daily meals (breakfast, lunch, or dinner) was changed to very low-calorie diet during preoperative 20 days [9]. However, careful consideration of this type of intervention is needed because malnutrition and sarcopenia are significant unfavorable prognostic predictors for survival in gastric cancer [31, 32]. Cho et al. conducted a trial of preoperative exercise therapy without diet control for reduction of visceral fat mass in patients with metabolic syndrome undergoing gastrectomy for early gastric cancer and found that postoperative morbidity was decreased by preoperative exercise. The training program comprised the following three components: aerobic exercise (such as the use of a treadmill or bicycle ergometer, swimming, dancing, or jogging), resistance training, and stretching during preoperative 28 days [8]. However, low BMI (<18.5 kg/m^2^) and visceral fat (<53.6 cm^2^) have been associated with a poor prognosis in patients with upper gastrointestinal cancer [26, 33]. Therefore, postoperative complications and long-term outcomes both require consideration in the development of an effective preoperative intervention for obese patients with gastric cancer.

The effects of several confounding factors, such as high BMI and sarcopenia, on surgical outcomes were evaluated in the present study; however, these were not significant [1, 4, 22, 24, 34–42]. BMI is calculated simply using the height and weight, and BMI ≥25 or ≥30 kg/m^2^ is widely used as a cutoff for obesity. In Japan, a wide variation in the VFA is found in the obese population with BMI ≥25 kg/m^2^ [43], and many studies have concluded that VFA is a better predictor of postoperative complications than BMI [3–5, 44]. In addition, CT is a precise method for measuring visceral fat [45] and is routinely performed preoperatively. This is convenient and has no extra associated costs or radiation exposure.

Sarcopenia has gained attention recently as a predictive marker for postoperative complications and prognosis in patients with many types of cancer, including gastric cancer [32, 44, 46–48]. The combination of sarcopenia and obesity, termed “sarcopenic obesity,” is recognized as a critical public health risk in an aging society. Sarcopenia and obesity share several pathophysiological mechanisms and may potentiate each other [49]. We have previously reported that sarcopenic obesity is a risk factor for SSI after laparoscopic total gastrectomy [4], and sarcopenic obesity was also a significant risk factor for infectious complications after laparoscopic total gastrectomy in this study (Tables [Supplementary-material supplementary-material-1] in the Supplementary Material). However, sarcopenia was not associated with the incidence of all complications after stomach resection, including distal gastrectomy. Although we conducted post hoc analysis using other cutoff values to define sarcopenia [50], there were no differences in the postoperative outcomes between patients with and without sarcopenia (data not shown). The clinical significance of this new biomarker requires further analysis.

The mechanism through which visceral obesity causes complications after gastrectomy is unclear; however, two hypotheses can be proposed. First, high visceral fat may be associated with technical difficulties in surgery. For example, false recognition of anatomy and excessive countertraction or pancreatic compression in lymph node dissection may increase the chance of injuring the pancreatic tissue [51–53]. Our study showed that excessive visceral fat was associated with a longer operating time and increased intraoperative blood loss. Excessive visceral fat was also associated with the development of abdominal abscess and pancreatic fistula that are closely related to operative manipulations [5]. We believe that this is the main mechanism through which visceral obesity causes complications. Second, excess visceral fat storage might impair the response to operative stress. Central obesity is known to affect adipocytokine-related inflammation and increase insulin resistance [24, 54–56], and these are the key processes that trigger surgical complications. These speculative ideas require examination in a future study.

There are certain limitations of this study. First, there may be selection and information biases due to the retrospective design. We excluded 33 patients (about 5% of the total study population) due to lack of availability of preoperative CT scan data; this may have created a selection bias. We minimized these biases by using information for consecutive patients taken from a prospectively maintained database. We also blinded the assessor of VFA to reduce bias. Second, there may have been unmeasurable confounding factors. However, we included almost all the major known risk factors for complications, including BMI, sarcopenia, diabetes mellitus, low albumin, total gastrectomy, and D2 lymphadenectomy; thus, we believe that this minimized the effect of the unmeasurable confounders. Third, the study was conducted at a single Japanese medical center. Asian populations are more prone to central obesity with increased insulin resistance compared to Caucasians [57, 58]; therefore, the reproducibility of the results requires evaluation in other medical centers and racial groups. Fourth, patients who underwent preoperative CT within two months (more than one month) before the surgery were included. However, most patients underwent CT within one month or 45 days before the surgery. Additionally, in patients undergoing neoadjuvant chemotherapy, preoperative CT was performed several weeks after the final administration. There may not be significant differences between body fat at the time of the CT and surgery. In fact, there was no significant difference between the body weight at the day of the preoperative CT and the body weight at few days before the surgery in patients who underwent CT more than 30 days before the surgery (difference = -0.21 ± 0.45 kg (mean ± SD), *P*=0.42). Thus, the period between the performance of CT and surgery might not significantly affect the results of this study.

## 5. Conclusions

The incidence of postoperative complications after gastrectomy increased in proportion to an increase in the preoperative VFA. To our knowledge, this is the first study to show the impact of VFA treated as a continuous variable on the complications following gastrectomy. Reduction in the VFA can be achieved with short-term exercise and nutrition therapy; however, the safety and effectiveness of this preoperative intervention remain unclear. We believe that the present study can serve as a stepping stone to improve the surgical outcomes in patients with gastric cancer.

## Figures and Tables

**Table 1:** Characteristics of the patients.

Variable	Visceral fat area (cm^2^)				*P* value
	0–49 *n* = 91	50–99 *n* = 160	100–149 *n* = 187	≥150 *n* = 128	
Age					
Continuous	64.8 ± 13.9	66.8 ± 11.1	67.3 ± 8.9	69.8 ± 9.4	0.0065
Sex					
Male	36 (39.6%)	92 (57.5%)	129 (69.0%)	114 (89.1%)	<0.0001
BMI (kg/m^2^)					
≥25	0 (0%)	13 (8.1%)	46 (24.6%)	72 (56.3%)	<0.0001
Subcutaneous fat (cm^2^)					
Continuous	58.6 ± 5.0	112 ± 3.8	133 ± 3.5	153 ± 4.2	<0.0001
ASA score					
≥3	4 (4.4%)	14 (8.8%)	14 (7.5%)	19 (14.8%)	0.042
DM					
Yes	3 (3.3%)	13 (8.1%)	21 (11.2%)	31 (24.2%)	<0.0001
Sarcopenia					
Yes	49 (53.9%)	114 (71.3%)	117 (62.6%)	74 (62.5%)	0.025
NAC					
Yes	18 (19.8%)	17 (10.6%)	28 (15.0%)	8 (6.3%)	0.015
cStage					
I	58 (63.7%)	110 (68.8%)	115 (61.5%)	81 (63.3%)	0.035
II	11 (12.1%)	24 (15.0%)	39 (20.9%)	33 (25.8%)	
III	22 (24.2%)	26 (16.3%)	33 (17.7%)	14 (10.9%)	
Serum albumin (g/dL)					
≤3.5	23 (25.3%)	28 (17.5%)	25 (13.4%)	14 (10.9%)	0.023
Operative procedure					
DG	61 (67.0%)	120 (75.0%)	129 (69.0%)	83 (64.8%)	0.28
TG	30 (33.0%)	40 (25.0%)	58 (31.0%)	45 (35.2%)	
Surgical approach					
Laparoscopic	83 (91.2%)	146 (91.3%)	177 (94.7%)	105 (82.0%)	0.0028
Open	2 (2.2%)	2 (1.3%)	7 (3.7%)	7 (5.5%)	
Robotic	6 (6.6%)	12 (7.5%)	3 (1.6%)	16 (12.5%)	
Lymph node dissection					
D1+	43 (47.3%)	75 (46.9%)	90 (48.1%)	64 (50.0%)	0.96
D2	48 (52.7%)	85 (53.1%)	97 (51.9%)	64 (50.0%)	

BMI: body mass index; ASA: American Society of Anesthesiologists; DM: diabetes mellitus; NAC: neoadjuvant chemotherapy; TG: total gastrectomy; DG: distal gastrectomy.

**Table 2:** Details of postoperative complications.

Postoperative complications	Visceral fat area (cm^2^)				*P* value
	0–49 *n* = 91	50–99 *n* = 160	100–149 *n* = 187	≥150 *n* = 128	
Abdominal abscess	2 (2.2%)	8 (5.0%)	16 (8.6%)	21 (16.4%)	0.0005
Pancreatic fistula	0 (0%)	1 (0.6%)	8 (4.3%)	8 (6.3%)	0.0089
Anastomotic leakage	2 (2.2%)	5 (3.1%)	4 (2.1%)	6 (4.7%)	0.59
Pneumonia	1 (1.1%)	3 (1.9%)	3 (1.6%)	4 (3.1%)	0.71
Small bowel obstruction	1 (1.1%)	1 (0.6%)	0 (0%)	5 (3.9%)	0.016
Delayed gastric emptying	2 (2.2%)	3 (1.8%)	0 (0%)	1 (0.8%)	0.24
Wound infection	0	1 (0.6%)	2 (1.1%)	0	0.53
Others	3 (3.3%)	6 (3.8%)	10 (5.4%)	14 (10.9%)	0.037

**Table 3:** Univariate and multivariate analyses of complications.

Variable	Univariate analysis		Multivariate analysis	
	OR (95% CI)	*P* value	OR (95% CI)	*P* value
Visceral fat area				
Per 1 cm^2^	1.010 (1.007–1.014)	0.0003	1.009 (1.004–1.013)	0.0002
Operative procedure				
TG	1.84 (1.21–2.80)	0.0038	1.79 (1.15–2.79)	0.010
Age				
Per 1 year	1.031 (1.010–1.053)	0.0040	1.023 (0.99–1.047)	0.059
Sex				
Male	2.52 (1.55–4.10)	0.0001	1.61 (0.94–2.77)	0.083
BMI (kg/m^2^)				
≥25	1.64 (1.05–2.58)	0.029	0.96 (0.53–1.73)	0.89
ASA score				
≥3	2.40 (1.31–4.41)	0.0037	1.87 (0.97–3.62)	0.061
Diabetes mellitus				
Yes	1.78 (1.02–3.11)	0.042	1.07 (0.58–1.98)	0.82
Sarcopenia				
Yes	1.40 (0.91–2.15)	0.12	1.28 (0.78–2.10)	0.34
cStage				
II	1.52 (0.91–2.51)	0.13	1.18 (0.69–2.02)	0.55
III	1.45 (0.85–2.46)	0.17	1.32 (0.74–2.35)	0.34
Subcutaneous fat (cm^2^)				
Per 1 cm^2^	1.002 (0.99–1.006)	0.25		
Serum albumin (g/dL)				
≤3.5	1.32 (0.78–2.23)	0.29	—	—
NAC				
Yes	0.89 (0.48–1.66)	0.72	—	—
Surgical approach				
Open	1.40 (0.49–4.02)	0.53	—	—
Robotic	0.71 (0.29–1.73)	0.45	—	—
Lymph node dissection				
D2	1.30 (0.87–1.95)	0.21	—	—

OR: odds ratio; CI: confidence interval; BMI: body mass index; ASA: American Society of Anesthesiologists; NAC: neoadjuvant chemotherapy; TG: total gastrectomy.

## Data Availability

The availability of the clinical data used in this study is restricted by the Ethics Committee of Kyoto University Hospital to protect patient privacy. Data are available from K. Okada (email: kokada@kuhp.kyoto-u.ac.jp) for researchers who meet the criteria for access to confidential data.

## Supplementary Materials

Supplementary Materials Univariate and multivariate analyses of postoperative hospital stay, operation time, and blood loss during surgery are shown in Supplemental Tables 1–3. VFA was not significantly associated with length of postoperative hospital stay in multivariate analysis (regression coefficient (*β*): 0.008, 95% CI: −0.010–0.027, *P*=0.38). However, VFA was an independent predictor of the operating time (*β*: 0.23, 95% CI: 0.11–0.35, *P* < 0.001) and amount of blood loss (*β*: 0.43, 95% CI: 0.12–0.73, *P*=0.006). Stratified analyses for BMI and DM are shown in Supplemental Tables 4 and 5. These analyses also showed significant relationships between VFA and postoperative complications. The relationship between sarcopenic obesity and infectious complications after laparoscopic total gastrectomy is shown in Supplemental Table 6. Sarcopenic obesity was a significant risk factor for infectious complications after laparoscopic total gastrectomy (*P*=0.049).
