# Supplementary material for: The Incidence of Postoperative Complications after Gastrectomy Increases in Proportion to the Amount of Preoperative Visceral Fat
Source: J Oncol. 2019 Dec 16;2019:8404383. doi: 10.1155/2019/8404383 (PMC6942899; doi:10.1155/2019/8404383)
Supplement: Supplementary Materials — Univariate and multivariate analyses of postoperative hospital stay, operation time, and blood loss during surgery are shown in Supplemental Tables 1–3. VFA was not significantly associated with length of postoperative hospital stay in multivariate analysis (regression coefficient (β): 0.008, 95% CI: −0.010–0.027, P=0.38). However, VFA was an independent predictor of the operating time (β: 0.23, 95% CI: 0.11–0.35, P < 0.001) and amount of blood loss (β: 0.43, 95% CI: 0.12–0.73, P=0.006). Stratified analyses for BMI and DM are shown in Supplemental Tables 4 and 5. These analyses also showed significant relationships between VFA and postoperative complications. The relationship between sarcopenic obesity and infectious complications after laparoscopic total gastrectomy is shown in Supplemental Table 6. Sarcopenic obesity was a significant risk factor for infectious complications after laparoscopic total gastrectomy (P=0.049). [file 8404383.f1.pdf]

Supplementary Materials

Univariate and multivariate analyses of postoperative hospital stay, operation time and blood loss during surgery are shown in Supplemental Tables 1-3. VFA was not significantly associated with length of postoperative hospital stay in multivariate analysis (regression coefficient [β] 0.008, 95% CI -0.010–0.027, P=0.38). However, VFA was an independent predictor of the operating time (β 0.23, 95%CI 0.11–0.35, P<0.001) and amount of blood loss (β 0.43, 95% CI 0.12–0.73, P=0.006).

Supplemental Table 1: Univariate and multivariate analyses of postoperative hospital stay

Variable		Univariate analysis		Multivariate analysis	
		Regression coefficient [β] (day) (95% CI)	P value	Regression coefficient [β] (day) (95% CI)	P value
Visceral fat area	per 1 cm^2	0.025 (0.009 – 0.040)	0.002	0.008 (-0.010 – 0.027)	0.38
Operative procedure	TG	2.44 (1.48 – 3.39)	<0.001	2.35 (1.41 – 3.29)	<0.001
Age	per 1 year	0.14 (0.053 - 0.22)	0.0014	0.11 (0.026 – 0 20)	0.011
Surgical approach	open	5.60 (4.18 – 7.03)	0.012	3.65 (1.32 – 6.17)	0.023
	robotic	1.51 (-0.63 – 3.64)	0.17	0.58 (-1.50 – 2.66)	0.59
Sex	male	0.81 (-0.14 – 1.75)	0.093	0.35 (-0.61 – 1.32)	0.47
BMI (kg/m^2)	≥25	1.33 (0.27 – 2.39)	0.014	1.05 (-0.15 – 2.24)	0.085
ASA score	≥3	3.10 (-0.03 – 6.23)	0.052	1.97 (-1.14 – 5.09)	0.21
Lymph node dissection	D2	1.55 (-0.25 – 3.35)	0.091	1.13 (-0.82 – 3.08)	0.25
cStage	II	3.26 (0.93 – 5.59)	0.006	1.73 (-0.70 – 4.17)	0.16
	III	3.24 (0.85 - 5.12)	0.007	0.90 (-3.12 – 6.32)	0.54
Serum albumin (g/dl)	≤3.5	2.40 (-0.052 – 4.85)	0.055	1.42 (-1.23 – 4.06)	0.29
Diabetes mellitus	yes	0.47 (-0.91 – 1.86)	0.50	-	-

Sarcopenia	yes	0.42 (-0.51 – 1.35)	0.38	-	-
NAC	yes	0.32 (-1.04 – 1.68)	0.64	-	-

BMI: body mass index, ASA: American Society of Anesthesiologists, NAC: neoadjuvant chemotherapy, TG: total gastrectomy

Supplemental Table 2: Univariate and multivariate analyses of operation time

Variable		Univariate analysis		Multivariate analysis	
		Regression coefficient [β] (min) (95% CI)	P value	Regression coefficient [β] (min) (95% CI)	P value
Visceral fat area	per 1 cm^2	0.36 (0.23 – 0.49)	<0.001	0.23 (0.11 – 0.35)	<0.001
Operative procedure	TG	49.6 (42.4 – 56.7)	<0.001	43.7 (37.7 – 49.6)	<0.001
NAC	yes	38.1 (27.0 – 49.1)	<0.001	12.2 (1.5 – 22.8)	0.025
Sex	male	16.3 (8.4 – 24.1)	<0.001	8.5 (2.4 – 14.6)	0.007
BMI (kg/m^2)	≥25	19.1 (10.2 – 27.9)	<0.001	10.8 (3.3 – 18.3)	0.005
Surgical approach	open	-24.2 (-35.8 – -12.7)	0.008	-59.8 (-72.6 – -45.6)	<0.001
	robotic	26.9 (9.6 – 44.3)	0.002	10.5 (-2.7 – 23.6)	0.12
Lymph node dissection	D2	56.4 (41.9 – 70.8)	<0.001	30.1 (17.8 – 42.4)	<0.001
cStage	II	43.5 (24.8 – 62.3)	<0.001	21.9 (6.3 – 37.6)	0.006
	III	78.5 (35.7 – 121.2)	<0.001	38.9 (15.4 – 52.3)	0.004
Serum albumin (g/dl)	≤3.5	22.6 (1.9 – 43.3)	0.033	17.8 (1.5 – 34.1)	0.033
Age	per 1 year	0.050 (-0.66 – 0.76)	0.89	-	-
ASA score	≥3	-15.9 (-42.4 – 10.6	0.24	-	-
Diabetes mellitus	yes	5.8 (-5.9 – 17.5)	0.33	-	-
Sarcopenia	yes	-1.54 (-9.39 – 6.31)	0.70	-	-

BMI: body mass index, ASA: American Society of Anesthesiologists, NAC: neoadjuvant chemotherapy, TG: total gastrectomy

Supplemental Table 3: Univariate and multivariate analyses of blood loss during surgery

Variable		Univariate analysis		Multivariate analysis	
		Regression coefficient [β] (ml) (95% CI)	P value	Regression coefficient [β] (ml) (95% CI)	P value
Visceral fat area	per 1 cm^2	0.63 (0.31 – 0.95)	<0.001	0.43 (0.12 – 0.73)	0.006
Operative procedure	TG	33.3 (13.3 – 53.3)	0.0038	25.4 (7.5 – 43.2)	0.006
Surgical approach	open	575 (549 - 602)	<0.001	558 (532 – 585)	<0.001
	robotic	194 (153 - 233)	<0.001	182 (142 – 221)	<0.001
Lymph node dissection	D2	54.5 (17.5- 91.5)	0.004	54.9 (18.4 – 91.4)	0.003
Sex	male	19.8 (0.26 – 39.3)	0.047	8.6 (-9.7 – 26.9)	0.35
NAC	yes	29.9 (1.9 – 57.9)	0.037	8.1 (-23.4 – 39.5)	0.61
cStage	II	39.7 (-8.7 – 88.1)	0.11	-23.2 (-69.2 – 22.7)	0.32
	III	61.1 (19.8 – 103.4)	0.012	-14.2 (-48.7 – 30.8)	0.34
Age	per 1 year	-0.060 (-1.80 – 1.68)	0.95	-	-
BMI (kg/m^2)	≥25	11.4 (-10.6 – 33.5)	0.31	-	-
ASA score	≥3	10.9 (-54.1 – 75.9)	0.74	-	-
Diabetes mellitus	yes	-6.12 (-34.7 – 22.5)	0.67	-	-
Sarcopenia	yes	-9.3 (-28.5 - 9.9)	0.34	-	-
Serum albumin (g/dl)	≤3.5	22.7 (-28.2 – 73.5)	0.38	-	-

BMI: body mass index, ASA: American Society of Anesthesiologists, NAC: neoadjuvant chemotherapy, TG: total gastrectomy

Supplemental Table 4: Stratified analysis about BMI

BMI<25 (n=435)

		OR (95% CI)	P value
Visceral fat area	per 1 cm^2	1.009 (1.004 – 1.015)	0.001

BMI≥25 (n=131)

		OR (95% CI)	P value
Visceral fat area	per 1 cm^2	1.009 (1.002 – 1.017)	0.016

Supplemental Table 5: Stratified analysis about DM

DM : no (n=498)

		OR (95% CI)	P value
Visceral fat area	per 1 cm^2	1.009 (1.005 – 1.014)	<0.001

DM : yes (n=68)

		OR (95% CI)	P value
Visceral fat area	per 1 cm^2	1.009 (0.91 – 1.077)	0.097

Supplemental Table 6: Relationship between sarcopenic obesity (SO) and infectious complications after laparoscopic total gastrectomy (n=153)

	SO (n=41)	The other (n=112)	P value
Infectious complications	12 (29%)	17 (15%)	0.049

SO are patients with sarcopenia (using Prado's criteria) and obesity (VFA≥100 cm^2).

Infectious complications include superficial SSI, abdominal abscess, pancreatic fistula and anastomotic leakage that are grade II or higher using the Clavien-Dindo classification.
